# Tree foliage as a net accumulator of highly toxic methylmercury

**DOI:** 10.1038/s41598-024-51469-x

**Published:** 2024-01-19

**Authors:** Idus Stinson, Han-Han Li, Martin Tsz-Ki Tsui, Peijia Ku, Yener Ulus, Zhang Cheng, Hon-Ming Lam

**Affiliations:** 1 Department of Biology, University of North Carolina at Greensboro, Greensboro, NC 27402 USA; 2 School of Life Science and Engineering, Southwest University of Science and Technology, Mianyang, 621010 China; 3 School of Life Sciences, State Key Laboratory of Agrobiotechnology, The Chinese University of Hong Kong, Shatin, New Territories Hong Kong SAR, China; 4 Institute of Environment, Energy and Sustainability, The Chinese University of Hong Kong, Shatin, New Territories Hong Kong SAR, China; 5 Environmental Sciences Division, Oak Ridge National Laboratory, Oak Ridge, TN 37830 USA; 6 Department of Environmental Studies, Davidson College, Davidson, NC 28035 USA; 7 College of Environment, Sichuan Agricultural University, Chengdu, 611130 China

**Keywords:** Environmental monitoring, Geochemistry

## Abstract

Tree canopies are known to elevate atmospheric inputs of both mercury (Hg) and methylmercury (MeHg). While foliar uptake of gaseous Hg is well documented, little is known regarding the temporal dynamics and origins of MeHg in tree foliage, which represents typically less than 1% of total Hg in foliage. In this work, we examined the foliar total Hg and MeHg content by following the growth of five individual trees of American Beech (*Fagus grandifolia*) for one growing season (April–November, 2017) in North Carolina, USA. We show that similar to other studies foliar Hg content increased almost linearly over time, with daily accumulation rates ranging from 0.123 to 0.161 ng/g/day. However, not all trees showed linear increases of foliar MeHg content along the growing season; we found that 2 out of 5 trees showed elevated foliar MeHg content at the initial phase of the growing season but their MeHg content declined through early summer. However, foliar MeHg content among all 5 trees showed eventual increases through the end of the growing season, proving that foliage is a net accumulator of MeHg while foliar gain of biomass did not “dilute” MeHg content.

## Introduction

Mercury (Hg) is a global pollutant that can essentially contaminate all natural and polluted environments, due mainly to its widespread sources, and long-range atmospheric transport and deposition^1^. Forested and vegetated landscapes are particularly prone to atmospheric Hg contamination^2^ because plant foliage can accumulate gaseous elemental Hg [*abbreviated as Hg(0)*] through the stomatal uptake^3^, and gaseous Hg(0) can be oxidized inside the foliar tissues to become inorganic Hg [*abbreviated as Hg(II)*] through binding to thiol groups^4^.

During the growing season, both the concentrations and total foliar mass of Hg(II) (as reflected by total Hg (*THg*) measurements) increase in the foliage, and its concentrations peak approximately when the foliage senesces^5–7^. Upon litterfall, foliar Hg(II) can be incorporated into the forest floor which can represent an input of atmospheric Hg to the forest floors, typically referred to dry deposition^2^. Subsequently, some of Hg(II) in the decomposing litter is released with dissolved organic matter (DOM) into water, and aqueous Hg(II) may be exported from the watershed through streamflow in the form of Hg-DOM complexes^8^. Once in the aquatic environment, litter-derived Hg(II) can be methylated in surface sediments in downstream streams, lakes, and wetlands by a range of anaerobic microbes possessing *hgcAB* two-gene clusters to produce highly toxic methylmercury [*abbreviated as MeHg*]^9^. In the aquatic environments, MeHg can extensively bioaccumulate and biomagnify in the natural food webs, leading to dangerously high levels in top aquatic predators and humans through fish consumption^10^.

In addition to the above “conventional” microbial pathways for the production of MeHg in the forested watersheds, MeHg production can potentially occur through different, but much less understood pathways. Indeed, MeHg can be widely detected, often in trace quantities, in different seemingly oxic environmental compartments in forests including air, precipitation, soils, and foliage. In the atmosphere, a few studies have reported low but measurable MeHg in precipitation^11–14^. Interestingly, fogwater may contain considerably more MeHg than rainwater (e.g., 3.4 ng/L in fogwater *vs.* 0.1 ng/L in rainwater, in coastal California, USA)^15^. However, there was some evidence of the existence of low-level MeHg in the gaseous phase^16^ in the range between 1 and 7 pg/m^3^, thus both gaseous and precipitation-derived MeHg may represent atmospheric sources to growing foliage. On the ground, tree foliage and litter also contain trace amounts of MeHg (e.g., < 0.2 ng/g dry wt) in forests distant from point sources^6,17–19^, and similar levels were observed in surface organic horizons of soil^17,18^. However, the exact sources of MeHg observed in growing foliage are poorly understood and constrained. Further, to our knowledge the temporal patterns of MeHg in growing foliage have not been examined; thus little is known about if and how MeHg changes in foliage over time during growing phase. Thus, both external sources (e.g., atmospheric uptake, or root uptake of soil solution) and internal sources (e.g., in-vivo methylation) are plausible pathways for MeHg accumulation in foliage^3,18^. Alternatively, microbial methylation of Hg(II) on the foliar surface is indeed feasible because a small amount of wet-deposited Hg(II) would be often present on foliar surfaces^7^ while microbial endophytic communities (regardless of anaerobes or not) may be often associated with leaf surface^20^, but it is unknown if these microbes can methylate Hg(II).

On the other hand, MeHg can be degraded (or demethylated) by both abiotic pathways (e.g., ultraviolet radiation in sunlight)^21^ and biotic pathways (e.g., microbes with *merB* gene)^22^, with both pathways relatively well studied in aquatic, but not in the terrestrial, ecosystems. Through measuring the stable Hg isotope signatures in the terrestrial food web components, empirical evidence of photodemethylation of MeHg has been demonstrated in upland forest ecosystems through the identification of positive mass-independent fractionation in MeHg^23^. However, the precise locations where the photodemethylation process in forests happens are not known.

In this study, given the paucity of information on MeHg dynamics in growing foliage, we aimed to fill this knowledge gap through examining the temporal changes of THg and MeHg in growing foliage, and attempted to relate their variations with other environmental factors (e.g., air temperature, rainfall, and evapotranspiration) as well as tree physicochemical parameters (e.g., leaf mass and area, stomatal density, and chlorophyll content). We regularly collected the foliar samples from five designated trees of American Beech in a forest of an urban campus in Greensboro, North Carolina, USA, and soil samples in the forest were collected at the beginning of the study to determine if soil Hg is elevated^24^. We predicted that foliar THg concentrations would increase with the growing season as demonstrated previously^5–7^, while we also predicted that foliar MeHg concentrations would increase over time since foliar THg has been shown to be positively correlated with that of MeHg among different tree species in a previous study^18^.

## Results and discussion

### Growing season and physicochemical characteristics

The bud break began around early to mid-April 2017 and litterfall started around mid-November 2017, resulting in a growing season of ~ 7 months. In comparison, the length of growing season in our study location (Greensboro, NC, USA) is considerably much longer than those of more northern environments in upstate New York, USA, in which two previous studies reported there were only ~ 4–5 months^5,6^, which indeed provided us a wider time window in observing the temporal changes of both THg and MeHg concentrations in foliar tissues.

During the ~ 7 months of growing season, the ambient weather changed temporally, and below is a summary of the overall changes of some key parameters as related to tree growth. First, the daily temperature increased from April through July from the monthly median value of 18.4 to 25.7 °C, but then it declined from August through November rapidly from the monthly median value of 25.7 to 9.1 °C (Supplemental Information (SI) Fig. S1A). Similarly, the monthly median value of daily solar radiation peaked around June and July (265.6 and 262.0 W/m^2^, respectively) (SI Fig. S1A) and then declined afterwards, which would be strongly correlated with the trend of monthly median daily temperature (*r*^2^ = 0.749; *graph not shown*).

The growing season started with increasing monthly rainfall from April to June (from 158.0 to 351.5 mm) and then declined through the summer until October (from 351.5 to 71.6 mm). In the autumn, there was a slightly wet period as evidenced from the increasing rainfall from October to November (from 71.6 to 173.7 mm) (SI Fig. S1B, SI Table S1). As related to temperature, the estimated evapotranspiration in general declined from August through November, with the monthly median daily evapotranspiration decreased from 5.42 to 1.57 mm (SI Fig. S1C).

Regarding the foliar samples, there were both temporal and tree-to-tree variations in physicochemical characteristics. Among the five trees studied, the individual leaf area appeared to be the smallest in the first sampling time point on 21 April (at 38.6 ± 5.8 cm^2^) but afterward the individual leaf area increased slightly and fluctuated between 40 and 50 cm^2^ (SI Fig. S2A, SI Table S2). Unlike individual leaf area, the individual leaf mass varied quite a bit over time among these five studied trees (SI Fig. S2B, SI Table S3). The averaged individual leaf mass increased from 0.22 ± 0.02 g on 21 April to a maximum of 0.43 ± 0.20 g on 16 June, but then the individual leaf mass gradually declined afterwards to 0.34 ± 0.05 g on 17 November (SI Fig. S2B, SI Table S3), which likely reflected some biochemical changes during the same period even though leaf size remained relatively constant throughout. By accounting for both individual leaf area and leaf mass, we calculated the foliar density (or leaf mass per unit area) and found that the foliar density was obviously low early on until mid-June, and afterwards the foliar density remained more or less stable until the litterfall (SI Fig. S2C, SI Table S4). We interpreted that the temporal changes in foliar density would reflect the development of epidermal cell walls and cuticle^25^, as well as other biochemical changes such as synthesis of chlorophyll (*see below*).

As related to gaseous uptake of Hg(0), we found that stomatal density remained quite similar throughout the growing season (note that we did not analyze this parameter for the first sampling on 21 April) (SI Fig. S3A, SI Table S5). Still, we found that the averaged stomatal density peaked in August (64,278 ± 12,888/cm^2^) but it was 20% higher than the average value found on 19 May (52,590 ± 6454/cm^2^). Lastly, the critical photosynthetic pigments, represented by total chlorophyll content (i.e., summation of Chl-a and Chl-b) appeared to increase from 21 April (mean: 0.80 ± 0.16 mg/g) through mid-June (between 2.85 and 3.19 mg/g), but after mid-September total chlorophyll content declined to the lowest right before litterfall at 0.50 ± 0.57 mg/g (SI Fig. S3B, SI Table S6). These total chlorophyll values were comparable but slightly lower than those reported for tree foliage along a large geographic gradient in China^26^. However, the averaged ratio of Chl-a/Chl-b remained quite constant (over 3.0) throughout the growing season, but the ratio in the fresh litter significantly dropped to 2.2 (*data not shown*). Overall, we did not find an apparent influence of the tree size (or age) imposed on these foliar physicochemical properties (i.e., Tree 2 *vs.* Tree 1, 3, 4, and 5).

### Foliar accumulation of total mercury

In the urban campus forest, analysis of soil samples showed a relatively low THg content (i.e., 73.6 ± 5.5 ng/g dry wt; *n* = 2) and the values are comparable to forests without point sources in the east coast of USA (e.g., 31–145 ng/g dry wt)^24^, indicating that there is no significant point source of Hg pollution in our study site and the majority of foliar Hg should be derived from non-soil pathway^3^.

**Figure 1:**
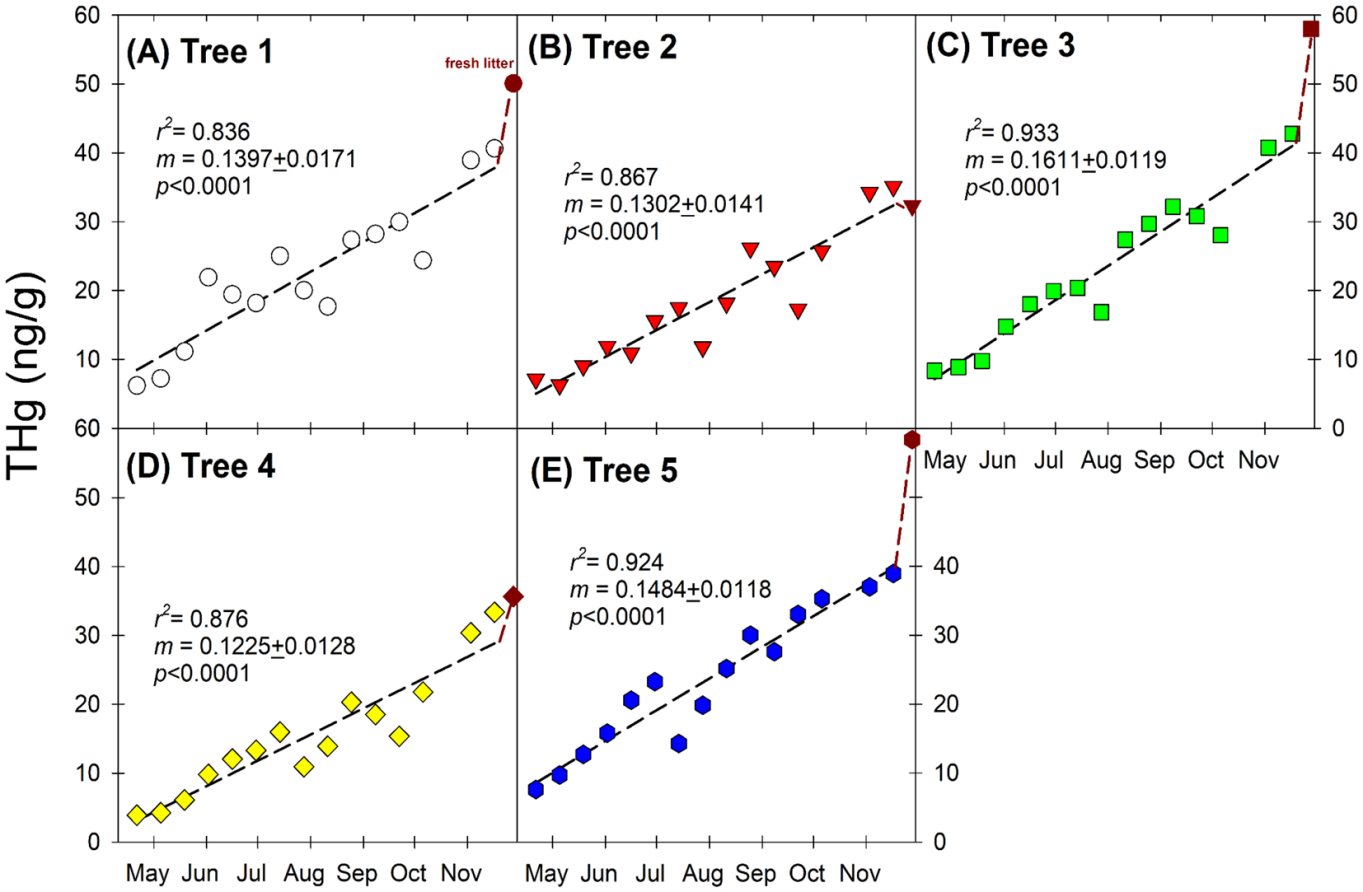
Temporal variations of total mercury (THg) concentrations in foliage and fresh litter in five American Beech trees over the growing season in the year of 2017. Linear regression analysis was performed for all foliar data (except the fresh litter data, shown in red color).

At the beginning of the growing season, the foliar tissues of the 5 trees started at very low THg content (mean ± S.D.: 5.8 ± 1.5 ng/g; range 3.4–6.7 ng/g) on 21 April (Fig. 1A–E, SI Table S7), which are similar to the beech trees reported in two previous studies in upstate New York at 4.9 ± 0.8 ng/g^5^ and 5.6 ± 3.6 ng/g^6^. Throughout the growing season, there was clearly a net accumulation of THg in foliar tissues over time, reaching 27.4 ± 4.6 ng/g by the end of the summer (25 August), and further reaching a maximum THg content in foliage on 17 November (46.0 ± 7.5 ng/g), and fresh litter collected on 28 November (41.8 ± 7.3 ng/g). The THg content in the fresh litter samples were similar to those observed in different temperate forests without point sources across the USA^24^.

The daily THg accumulation rates (i.e., the slopes in Fig. 1A–E) ranged from 0.123 (Tree 4) to 0.161 ng/g/day (Tree 3) in this study, which would be considerably lower than that of beech trees in upstate New York. Specifically, Bushey et al.^6^ reported an average daily THg accumulation rate of 0.47 ng/g/day (understory foliage), and in their study averaged THg content of foliage could reach a maximum of 68 ng/g from bud break in only 4 months, as opposed to a growing season of ~ 7 months in our study. The much lower daily accumulation rate of THg in our study trees would probably be attributed to various factors, but we speculated that lower gaseous Hg(0) concentrations^3^ as well as a higher rate of photoreduction of Hg(II)^27^ may be responsible for these discrepancies.

### Temporal dynamics of foliar methylmercury content

**Figure 2:**
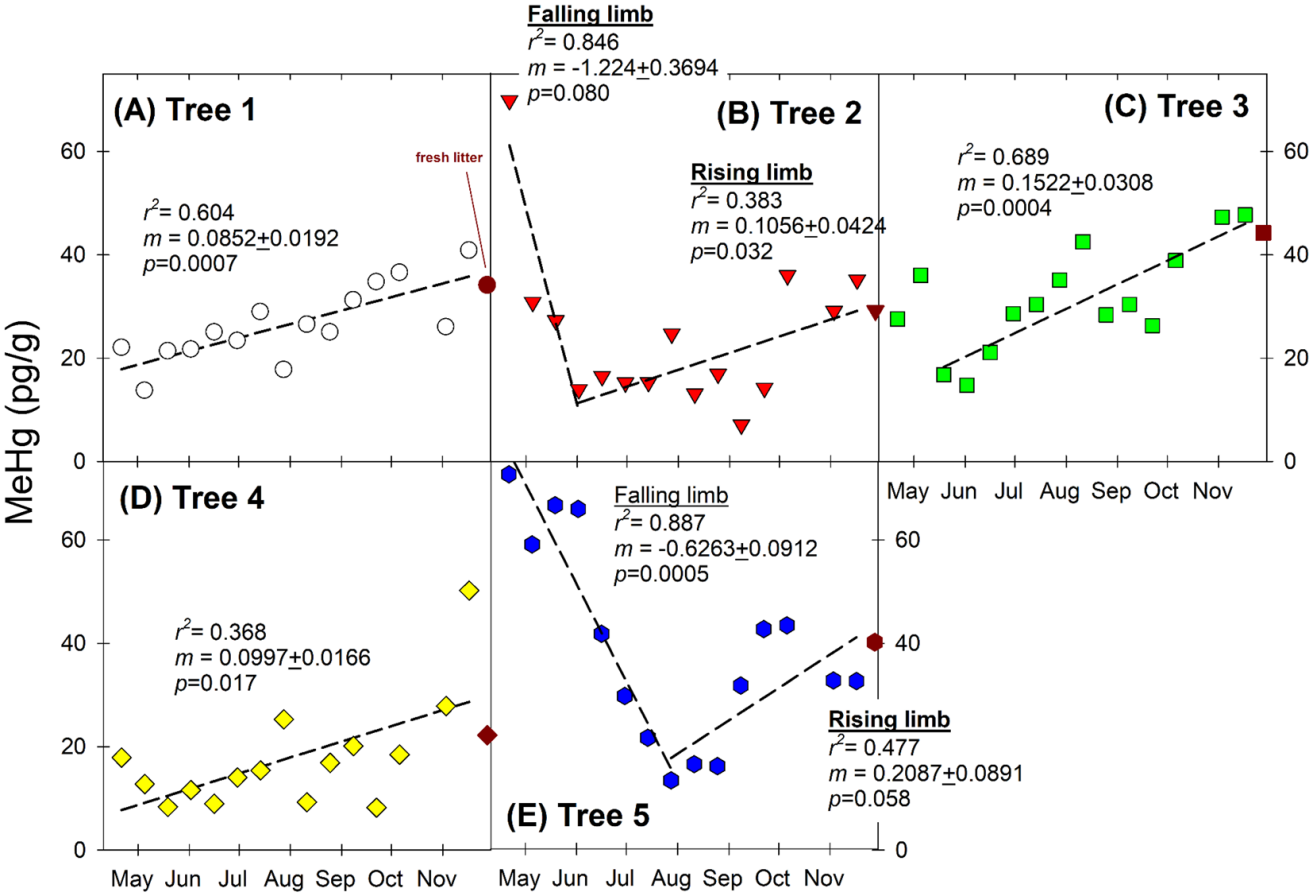
Temporal variations of methylmercury (MeHg) concentrations in foliage and fresh litter in five American Beech trees over the growing season in the year of 2017. Linear regression analysis was performed for all foliar data (except the fresh litter data, shown in red color).

From our five selected American Beech trees, the averaged MeHg content showed some temporal variations but did not show a continuous linear increase over the growing season as in the case of THg observed above for all five trees (Fig. 2A–E, SI Table S8). For the averaged values, MeHg content ranged from the lowest of 20.7 ± 5.7 pg/g on 25 August to the highest of 41.3 ± 7.6 pg/g on 17 November. However, there were apparent temporal fluctuations during the 6–7 months and there were also large tree-to-tree differences in MeHg content and thus reporting the averaged values would inadvertently mask these individual differences. Also, the percentage of THg as MeHg (i.e., %MeHg) averaged ~ 0.17%, which is lower than other forest studies^18,19^ and the underlying explanation is not known at the moment. It is interesting to note due to the lowest THg content for the initial foliar samples while their MeHg content was not necessarily the lowest, %MeHg changed over time in foliage. Apparently, we found that %MeHg was the highest for 21 April samples (0.96%) but %MeHg declined steadily afterward (SI Table S9).

By examining the MeHg data among individual trees (SI Table S8), it is noted that both Tree 2 and Tree 5 showed relatively high initial MeHg content (69.9 pg/g for Tree 2 and 72.7 pg/g for Tree 5 on 21 April, 2017) but both trees showed apparent declines of MeHg content. For Tree 2, the decline seemed to cease around mid-June but for Tree 5 the decline continued until late July (Fig. 2B,E). Interestingly, both Tree 2 and 5 showed upward trend of MeHg after the initial declines until litterfall, and with MeHg content reaching 29.2 pg/g for Tree 2 and 40.2 pg/g for Tree 5. In contrast, the trends appeared to be more straightforward for Tree 1, 3, and 4, in which MeHg content began with a slight small decline initially but their MeHg content all climbed steadily toward the highest levels before litterfall (Fig. 2A,C,D). If we only focused on the increasing trend of MeHg for all individual trees, the daily accumulation rate varied widely, ranging from 0.085 pg/g/day (Tree 1) to 0.209 pg/g/day (Tree 5). This analysis clearly showed the differences from the more consistent daily accumulation rate of THg for the five trees, showing the challenge in understanding the processes regulating MeHg accumulation in foliage.

### Potential factors influencing foliar methylmercury content

It appeared that both THg and MeHg content are very dynamic inside foliage as it grows. We attempted to evaluate if the weather and physicochemical parameters can explain their influences on THg and MeHg content but most of these parameters did not show any significant, direct influences. We found that if these parameters showing any statistically significant effects (*p* < 0.05) would be likely caused by their concurrent variations with seasonality or time (SI Tables S10, S11). For THg, we believe that the continuous uptake of gaseous Hg(0) would represent the best explanation of temporal increases of THg in foliage as revealed by previous studies^3,5–7^.

**Figure 3:**
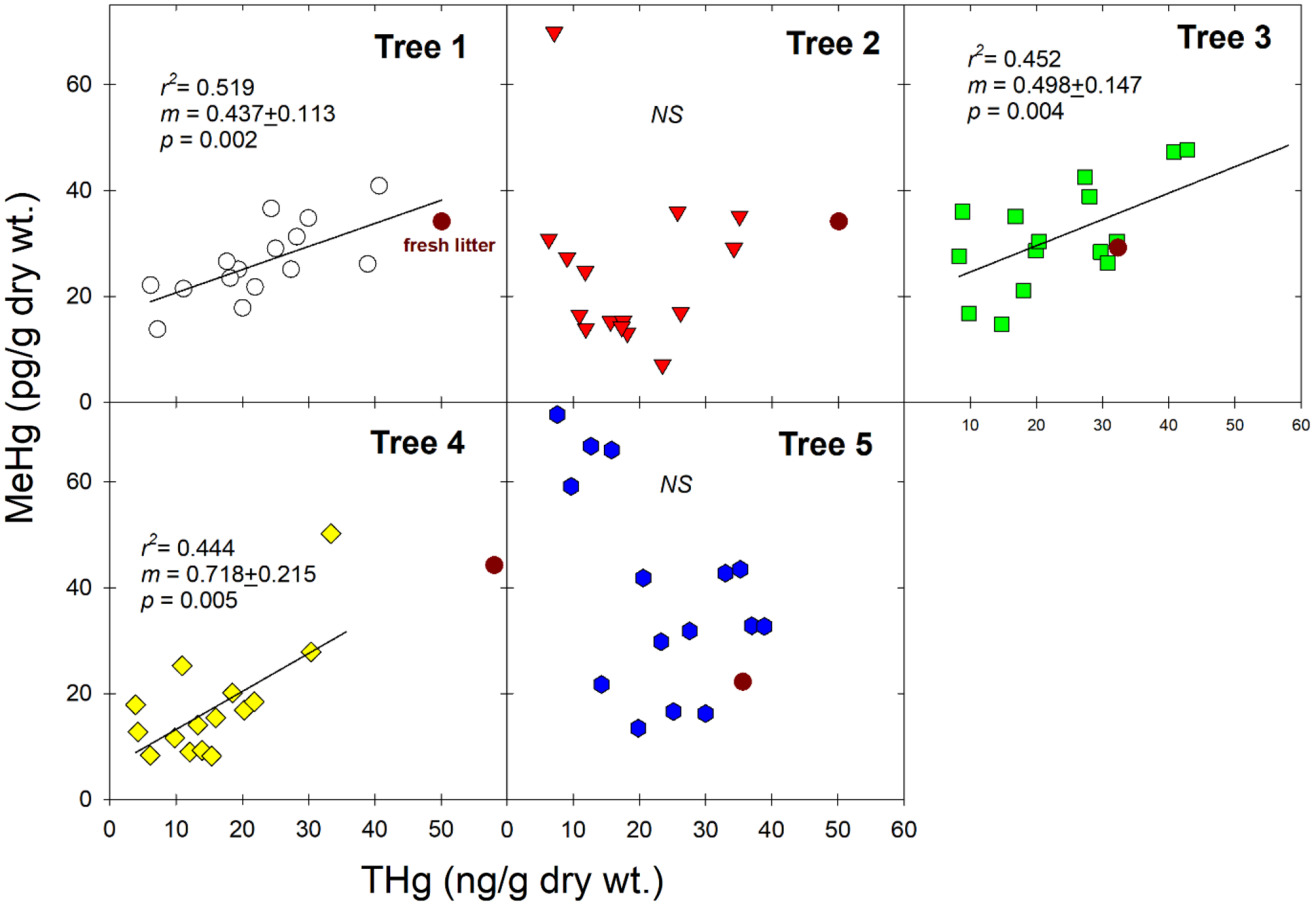
Relationship between THg and MeHg concentrations in foliage and fresh litter over the growing season. Linear regression was conducted only for understory foliage for each tree, and NS = no significant relationship was found.

In contrast, the potential explanations for the variations of MeHg can be numerous and here we provided a few potential mechanisms. First, the observations of strong association of THg and MeHg in foliage among different tree species prompted Tabatchnick et al.^18^ to hypothesize the in-vivo production of MeHg inside foliage, and the authors speculated the presence of common chemicals including vitamin B_12_, coniferol, acetate, and parahydroxybenzaldehyde would mediate and promote Hg(II) methylation abiotically^28,29^. Until now there has not been any direct empirical evidence to support or reject this hypothesis. From our study, while we also observed statistically significant relationships (*p* < 0.05) between THg and MeHg in foliar samples in Tree 1, 3, and 4, Tree 2 and 5 showed no statistically significant relationship due to the prolonged initial declines of MeHg (i.e., opposite trends of THg and MeHg during their respective initial stages) (Fig. 3). Thus, our data potentially supported more than one source(s) of MeHg in foliage, including the direct uptake of MeHg from precipitation^11–14^ and/or air^16^ in addition to potential in-vivo production^18^.

Second, the rapid initial declines of MeHg in Tree 2 and 5 suggested common mechanism(s) in reducing MeHg content. Since leaf mass and foliar density (SI Fig. S3) both increased steadily during the first 2 months of growth, we hypothesized that the development of epidermal cell walls and cuticle during early stages^25^ may actually allow more penetration of ultraviolet radiation (e.g., UV-B) that can mediate MeHg photodemethylation in foliar tissues^21^. Further, different foliar structures (e.g., anticlinal cell wall region) would also allow the penetration of UV-B in foliage even epidermal cell walls and cuticle are fully developed^30^. Recent studies using stable Hg isotopes demonstrated the photo-reductive loss of Hg(II) from living foliage and this represents a re-emission of Hg(0) from the trees^27^, which support the possibility of photodemethylation of MeHg inside living foliage.

Third, the mass biodilution pathway, which has been found to explain phytoplankton and animal’s declines of MeHg content upon rapid growth through processes such as eutrophication in lakes^31^, may seem to explain the initial declines of MeHg in Tree 2 and 5. However, while the leaf mass almost doubled in the first two months (SI Fig. S3), the total MeHg mass per individual leaf (not concentration) still declined strongly for both Tree 2 and 5. The increasing trend of MeHg mass per individual leaf persisted for Tree 1, 3, and 4, showing that the actual changes of MeHg (regardless of decrease or increase) is independent of the biomass gain (SI Fig. S4), thus rejecting the possibility of merely biomass dilution.

### Implications to methylmercury sources in forested ecosystems

It has long been known that foliage represents an important source of Hg(II) in forested landscapes, elevating the atmospheric deposition of Hg to these terrestrial ecosystems^2,3^. Also, the majority of previous studies focused on the subsequent transformation of Hg(II) to MeHg in the reducing environments especially aquatic habitats^32^. However, the often-overlooked pool of MeHg in growing foliage actually represents new inputs of MeHg to the forested environments^2,3^. This study, along with a few others^18^, showed that there must be complex yet not fully understood processes regulating the content of neurotoxic MeHg in foliage, and subsequently in fresh litter and the surface soils upon litterfall^17^. Our findings helped clarify that in growing tree foliage while THg concentrations increase linearly, MeHg concentrations and MeHg mass can change in different directions potentially due to production and demethylation occurring concurrently. Nevertheless, our data showed clearly that MeHg concentrations and MeHg mass per leaf increased overall during the growing season, pointing that the in-vivo production and/or external accumulation (e.g., from rainfall) should exceed the demethylation, if any.

## Methods

### Study site and sampling approach

**Figure 4:**
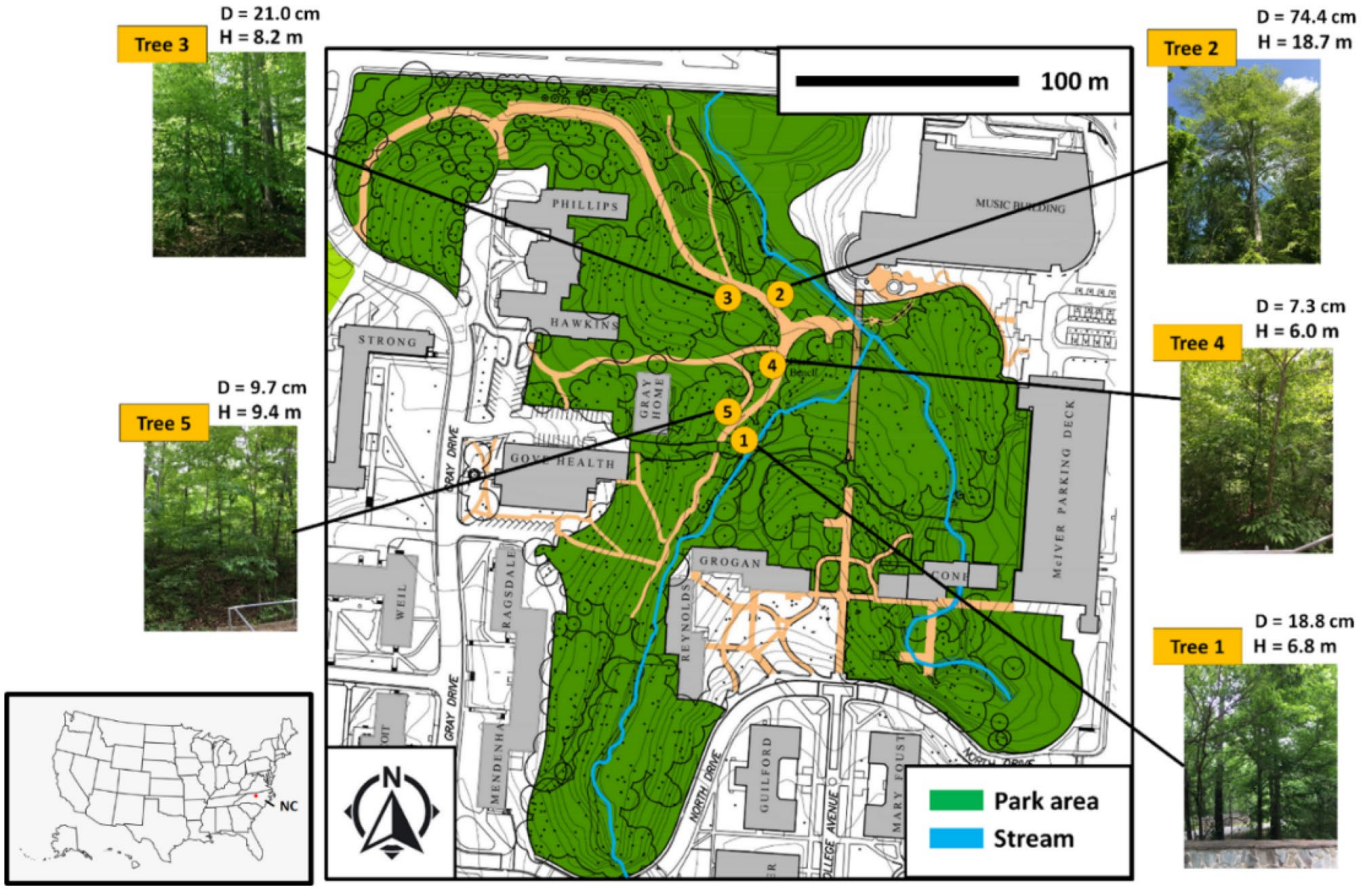
Map showing the positions of the five study Beech trees within the Peabody Park on the urban campus of the University of North Carolina at Greensboro (UNCG) at the city of Greensboro, North Carolina, USA. Also shown are the estimated tree height (H) in meters and tree trunk diameter (D) in centimeters. Source of map: www.uncg.edu.

This study was conducted by following the institutional guidelines of the University of North Carolina at Greensboro (UNCG), and the field sampling was conducted at the campus of UNCG (Greensboro, Guilford County, North Carolina, USA) (coordinates: 36°04'20.1"N 79°48'31.9"W) during a growing season from April to November, 2017. Our study did not involve species at the risk of extinction or trade of endangered species as identified by International Union for Conservation of Nature. Briefly, we selected five individual trees of American Beech (*Fagus grandifolia*) as the subjects of this study, and they were labeled as Tree 1, Tree 2, Tree 3. Tree 4, and Tree 5 (Fig. 4). It should be noted that the tree sizes were quite similar for all except Tree 2. For example, the tree height (H) ranged from 6.0 to 9.4 m for Tree 1, 3, 4, and 5 while that for Tree 2 was 18.7 m, similar differences were also observed for the tree trunk diameter (Fig. 4). American Beech was chosen because it is native to North Carolina and is common throughout the eastern USA, and has been examined for THg accumulation in at least two previous studies at upstate New York, USA^5,6^, allowing a comparison of our results with other studies.

After the foliage emerged in mid-April 2017, we conducted biweekly sampling until the litterfall in November 2017 (except missing one sampling point in mid-October). In brief, we used a pole pruner to collect 10–20 individual foliage on each side of four sides of the under canopy at each tree (i.e., a total of 40–80 foliage per tree was collected during each sampling) with personnel wearing powder-free vinyl gloves. Samples were carefully transferred into a new ziplock bag, and each of the five trees were sampled for a total of 15 times during the growing season in 2017, across the spring, summer, and autumn (i.e., 21 April, 5 May, 19 May, 2 June, 16 June, 30 June, 14 July, 28 July, 11 August, 25 August, 8 September, 22 September, 6 October, 3 November, and 17 November). It should be noted that on 28 November we collected freshly fallen, intact, litter on the ground, which can be derived from different heights of the tree (i.e., not restricted to understory). Also, we collected surface soil using a clean stainless-steel trowel at the beginning of the study to measure THg values. We retrieved the weather data (from March to December 2017) from a nearby (~ 7 km) weather station at the North Carolina A&T State University Research Farm (36°03'40.6"N 79°44'10.3"W; Greensboro, NC, USA).

### Measurements of physicochemical parameters and mercury content

We randomly picked foliage samples from each tree to measure for the physicochemical parameters. Specifically, we quantified individual foliage for leaf area (in cm^2^), leaf dry mass (in g), stomatal density (in stoma/cm^2^), and we used cold 80% methanol for extraction of foliar samples, followed by measurement by a spectrophotometer (following SERAS/USEPA SOP 2030) for quantifying chlorophyll-a and chlorophyll-b content (in mg/g; reported as total chlorophyll). In the laboratory, all remaining samples were used for Hg analysis, and they were frozen at − 20 °C and later lyophilized by a bench-top freeze-dryer (SP Scientific), and each sample was composited from foliage/litter at four directions of the tree and homogenized by a clean stainless-steel blender^33^.

For THg analysis, each homogenized sample was weighed into an acid-cleaned 40 mL borosilicate glass vial with PTFE-faced septum (Thermo Fisher Scientific), and completely digested with an aliquot of trace-metal grade concentrated HNO_3_ and H_2_O_2_ (4:1 v/v) (both from Thermo Fisher Scientific) at 80 °C overnight, reagent blank, and replicates of standard reference material (SRM) NIST-1515 (Apple Leaves) were included with each batch of sample digestion (QA/QC)^34^. To analyze for THg, an aliquot of acid digest was added to 100 mL ultrapure water in a glass bubbler, in which we added 200 µL of 30% NH_2_OH and then 200 µL of 20% SnCl_2_ to completely reduce Hg(II). We then purged the solution under a stream of Hg-free N_2_ gas for 15 min, and Hg(0) passed through a soda-lime trap and was collected by a gold trap downstream. Sample Hg was quantified by the double amalgamation technique with a cold vapor atomic fluorescence spectrometer (CVAFS, Brooks Rand Model III)^35^. Two certified reference standards at 1.00 ng/mL (SRM NIST-3133 and NIST-1641d) were used for developing the calibration curve, as described elsewhere^36^. We also included NIST-1515 (Apple Leaves) as the reference material to be included along the sample digestion and THg analysis, and the recovery averaged 97.9 ± 0.99% (*n* = 7).

For MeHg analysis, each homogenized sample was weighed into a new polypropylene centrifuge vial and added with 4.6 M trace-metal grade HNO_3_ (Thermo Fisher Scientific), and heated at 60 °C in a water bath for 12 h to release MeHg into the acid solution^37^. It should be noted that plant reference material certified for MeHg was not available during the time of this study, and our repeated analyses of SRM NIST-1515 Apple Leaves yielded a mean (± S.D.) of 54.01 ± 9.71 pg/g dry wt (*n* = 7). In brief, aliquot of the acid digest was added into 100 mL ultrapure water in a glass bubbler, in which we first neutralized the acidity with 4.6 M KOH, and MeHg was ethylated by the addition of 50 µL of ice-cold 1% NaBEt_4_ for 25 min. The solution was purged by a stream of Hg-free N_2_ gas for 12 min and ethylated Hg species was collected by a Tenax TA Trap (Supelco) downstream. The sample trap was dried by Hg-free N_2_ gas for 7 min before being heat-desorbed, and alkyl Hg species were separated by a gas chromatography and pyrolyzed at 700–800 °C^38^. MeHg was quantified by CVAFS (Brooks Rand Model III) and a standard solution at 1.00 ng/mL (supplied by CEBAM Analytical Laboratory; Bothell, Washington, USA) was used for developing the calibration curve. The actual MeHg concentration in the calibration standard was checked against our in-house THg standard (NIST-3133; 1 ng/mL) following a method outlined by USEPA^39^.

### Statistical analyses

We performed regression analyses by SigmaPlot 12.5 (Systat Software, Inc). The significance level for all statistical analyses was α = 0.05.

### Supplementary Information

Supplementary Information.

## Data Availability

All data generated or analysed during this study are included in this published article [and its Supplementary Information files].
