# Supplementary material for: Tree foliage as a net accumulator of highly toxic methylmercury
Source: Sci Rep. 2024 Jan 19;14:1757. doi: 10.1038/s41598-024-51469-x (PMC10799008; doi:10.1038/s41598-024-51469-x)
Supplement: Supplementary file 1 — Supplementary Information. [file 41598_2024_51469_MOESM1_ESM.docx]

***Supplemental Information (SI)***

**Tree foliage as a net accumulator of highly toxic methylmercury**

Idus Stinson ^a^, Han-Han Li ^b, *^, Martin Tsz-Ki Tsui ^a, c, d, *^, Peijia Ku ^a, e^, Yener Ulus ^a, f^, Zhang Cheng ^g^, Hon-Ming Lam ^c^

*^a^ Department of Biology, University of North Carolina at Greensboro, Greensboro, North Carolina 27402, USA*

*^b^ School of Life Science and Engineering, Southwest University of Science and Technology, Mianyang 621010, China*

*^c^ School of Life Sciences, State Key Laboratory of Agrobiotechnology, The Chinese University of Hong Kong, Shatin, New Territories, Hong Kong SAR, China*

*^d^ Institute of Environment, Energy and Sustainability, The Chinese University of Hong Kong, Shatin, New Territories, Hong Kong SAR, China*

*^e^ Environmental Sciences Division, Oak Ridge National Laboratory, Oak Ridge, Tennessee 37830, USA*

*^f^ Department of Environmental Studies, Davidson College, Davidson, NC 28035, USA*

*^g^ College of Environment, Sichuan Agricultural University, Chengdu 611130, China*

* Corresponding authors. H. Li ([lihanhan@swust.edu.cn](mailto:lihanhan@swust.edu.cn)) and M.T.-K. Tsui ([mtktsui@cuhk.edu.hk](mailto:mtktsui@cuhk.edu.hk))

**
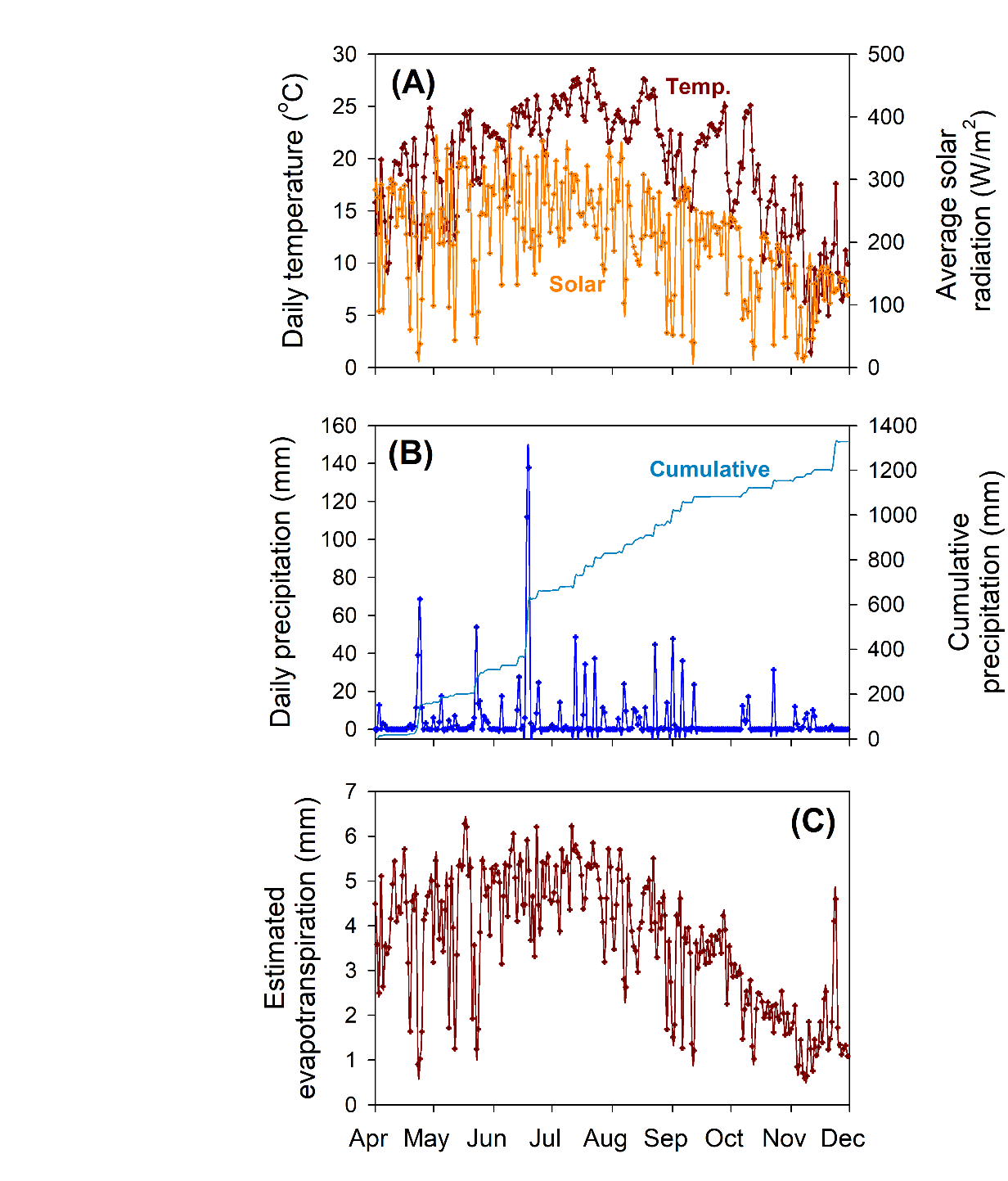
**

**Fig. S1** Ambient weather in the study area (Greensboro, North Carolina, USA) from 1 April through 30 November, 2017. **(A)** Daily temperature and solar radiation; **(B)** daily precipitation and cumulative precipitation; and **(C)** estimated evapotranspiration. The weather station is located at NC A&T Research Farm (36.061558, -79.736082) and data was retrieved from the North Carolina State Climate Office (USA).


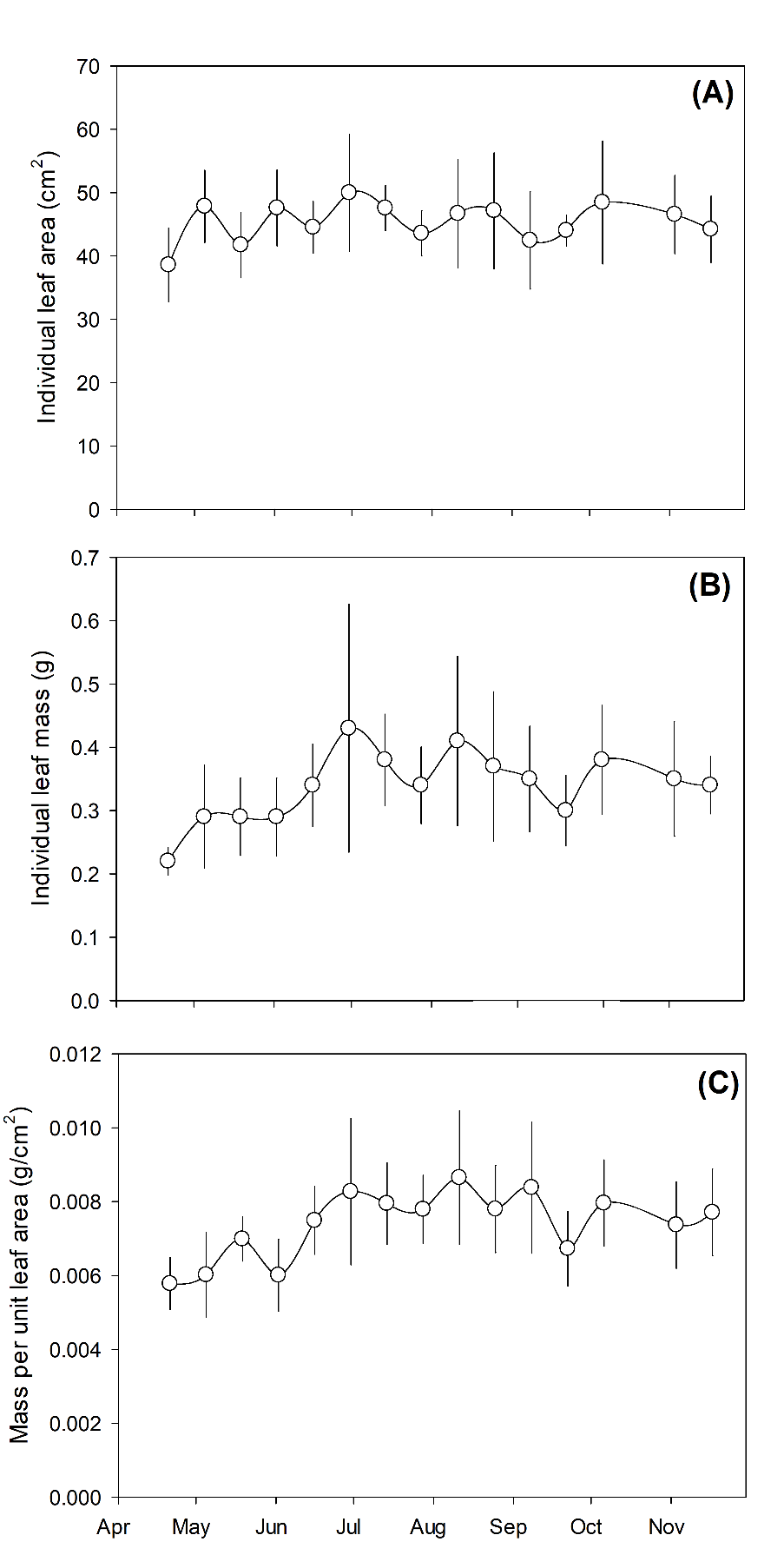


**Fig. S2** Temporal changes of foliar physicochemical characteristics, including **(A)** individual leaf area, **(B)** individual leaf mass, and **(C)** mass density of foliage.


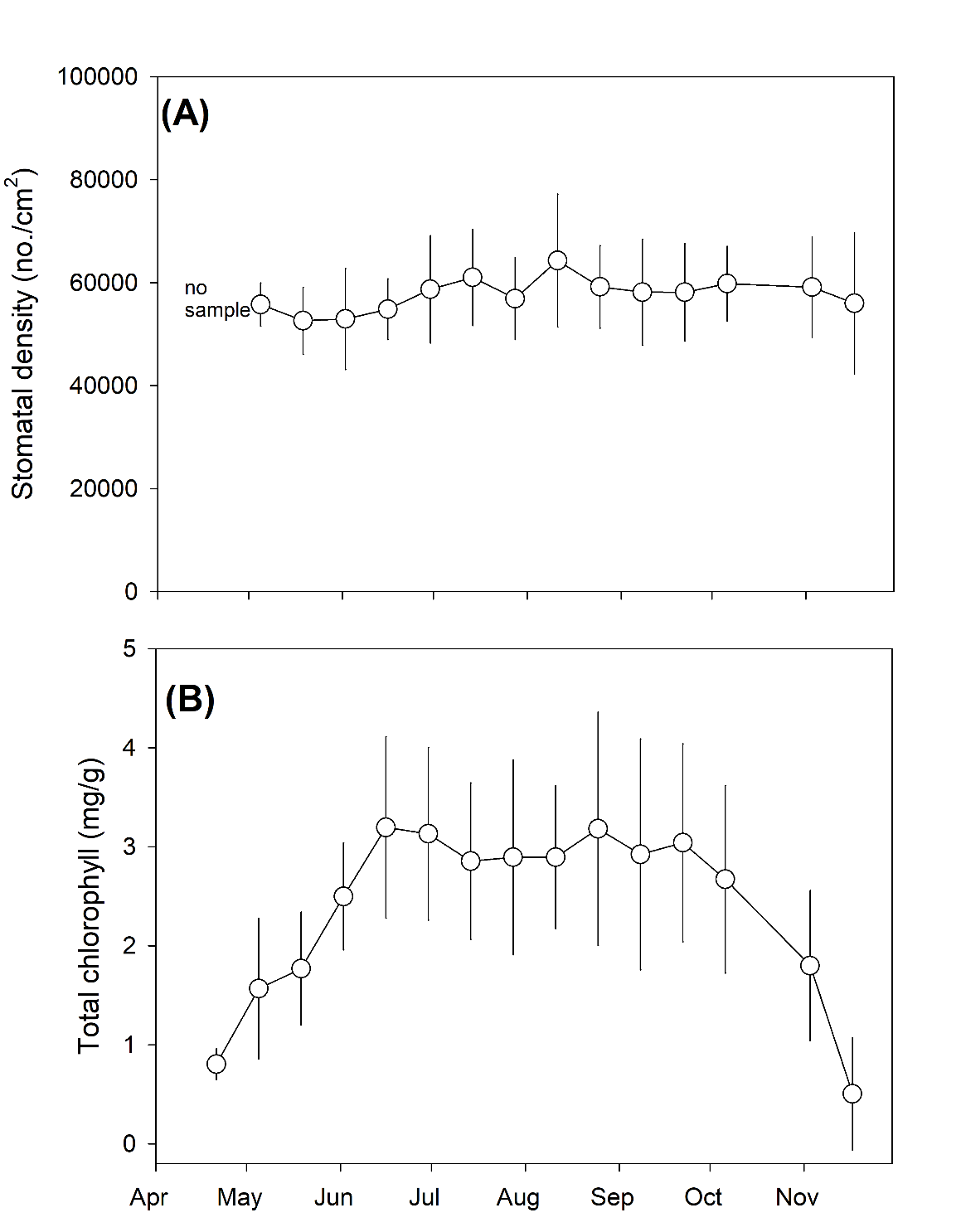


**Fig. S3** Temporal changes of foliar physicochemical characteristics, including **(A)** stomatal density, and **(B)** total chlorophyll content.


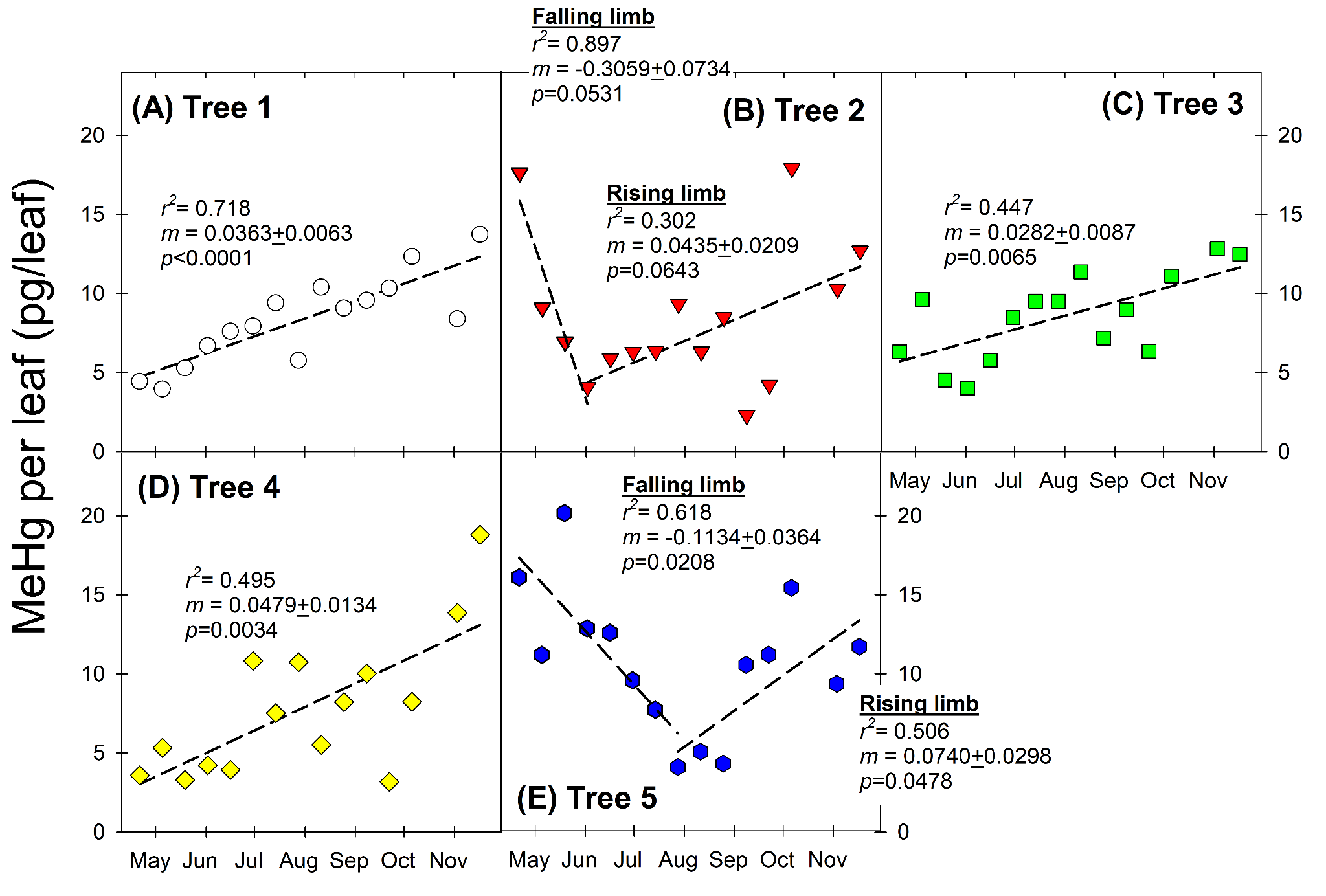


**Fig. S4** Temporal variations of total methylmercury (MeHg) mass per individual leaf over the growing season.

**Table S1** Monthly precipitation amount during the study period.

| **Month in 2017** | **Monthly precipitation amount (mm)** |
| --- | --- |
| Apr | 157.98 |
| May | 151.92 |
| Jun | 351.54 |
| Jul | 167.88 |
| Aug | 140.71 |
| Sep | 112.25 |
| Oct | 71.63 |
| Nov | 173.71 |

**Table S2** Leaf area of individual foliage (cm^2^) in this study.

| **Date** | **Type** | **Tree 1** | **Tree 2** | **Tree 3** | **Tree 4** | **Tree 5** |
| --- | --- | --- | --- | --- | --- | --- |
| 21 April, 2017 | Foliage | 36.54 | 48.20 | 32.64 | 36.54 | 38.97 |
| 5 May, 2017 | Foliage | 43.11 | 51.86 | 48.02 | 54.87 | 41.36 |
| 19 May, 2017 | Foliage | 38.20 | 36.97 | 41.69 | 50.20 | 41.72 |
| 2 June, 2017 | Foliage | 45.71 | 47.61 | 54.59 | 51.25 | 38.76 |
| 16 June, 2017 | Foliage | 41.26 | 49.07 | 42.41 | 48.85 | 41.07 |
| 30 June, 2017 | Foliage | 46.19 | 49.07 | 44.44 | 66.17 | 44.05 |
| 14 July, 2017 | Foliage | 43.15 | 50.89 | 47.97 | 51.07 | 44.71 |
| 28 July, 2017 | Foliage | 41.44 | 43.30 | 42.24 | 49.80 | 41.11 |
| 11 August, 2017 | Foliage | 42.26 | 58.26 | 42.33 | 52.94 | 37.76 |
| 25 August, 2017 | Foliage | 44.13 | 58.56 | 42.21 | 54.51 | 36.30 |
| 8 September, 2017 | Foliage | 38.25 | 42.96 | 48.63 | 50.67 | 31.84 |
| 22 September, 2017 | Foliage | 43.47 | 45.46 | 44.03 | 46.80 | 40.35 |
| 6 October, 2017 | Foliage | 39.39 | 64.99 | 44.50 | 46.76 | 46.66 |
| 3 November, 2017 | Foliage | 43.31 | 52.26 | 44.27 | 53.72 | 39.26 |
| 17 November, 2017 | Foliage | 36.34 | 49.91 | 43.06 | 47.94 | 43.88 |

**Table S3** Leaf mass of individual foliage (g) in this study.

| **Date** | **Type** | **Tree 1** | **Tree 2** | **Tree 3** | **Tree 4** | **Tree 5** |
| --- | --- | --- | --- | --- | --- | --- |
| 21 April, 2017 | Foliage | 0.2005 | 0.2525 | 0.2288 | 0.2005 | 0.2216 |
| 5 May, 2017 | Foliage | 0.2878 | 0.2953 | 0.2675 | 0.4164 | 0.1896 |
| 19 May, 2017 | Foliage | 0.2476 | 0.2545 | 0.2694 | 0.3964 | 0.3025 |
| 2 June, 2017 | Foliage | 0.3086 | 0.2935 | 0.2723 | 0.3637 | 0.1953 |
| 16 June, 2017 | Foliage | 0.3039 | 0.3577 | 0.2738 | 0.4385 | 0.3012 |
| 30 June, 2017 | Foliage | 0.3389 | 0.4130 | 0.2966 | 0.7695 | 0.3221 |
| 14 July, 2017 | Foliage | 0.3247 | 0.4147 | 0.3134 | 0.4881 | 0.3561 |
| 28 July, 2017 | Foliage | 0.3249 | 0.3775 | 0.2711 | 0.4248 | 0.3056 |
| 11 August, 2017 | Foliage | 0.3919 | 0.4831 | 0.2674 | 0.5957 | 0.3058 |
| 25 August, 2017 | Foliage | 0.3619 | 0.5017 | 0.2529 | 0.4870 | 0.2658 |
| 8 September, 2017 | Foliage | 0.3063 | 0.3230 | 0.2953 | 0.4984 | 0.3326 |
| 22 September, 2017 | Foliage | 0.2974 | 0.2966 | 0.2414 | 0.3878 | 0.2624 |
| 6 October, 2017 | Foliage | 0.3374 | 0.4978 | 0.2857 | 0.4461 | 0.3552 |
| 3 November, 2017 | Foliage | 0.3221 | 0.3536 | 0.2712 | 0.4973 | 0.2854 |
| 17 November, 2017 | Foliage | 0.3361 | 0.3616 | 0.2616 | 0.3744 | 0.3589 |

**Table S4** Foliar density of individual foliage (g/cm^2^) in this study.

| **Date** | **Type** | **Tree 1** | **Tree 2** | **Tree 3** | **Tree 4** | **Tree 5** |
| --- | --- | --- | --- | --- | --- | --- |
| 21 April, 2017 | Foliage | 0.005485 | 0.005238 | 0.007008 | 0.005485 | 0.005685 |
| 5 May, 2017 | Foliage | 0.006677 | 0.005694 | 0.005571 | 0.007588 | 0.004585 |
| 19 May, 2017 | Foliage | 0.006481 | 0.006883 | 0.006461 | 0.007897 | 0.007252 |
| 2 June, 2017 | Foliage | 0.006751 | 0.006165 | 0.004987 | 0.007096 | 0.005039 |
| 16 June, 2017 | Foliage | 0.007367 | 0.00729 | 0.006457 | 0.008977 | 0.007334 |
| 30 June, 2017 | Foliage | 0.007337 | 0.008416 | 0.006673 | 0.011628 | 0.007311 |
| 14 July, 2017 | Foliage | 0.007525 | 0.008149 | 0.006532 | 0.009556 | 0.007964 |
| 28 July, 2017 | Foliage | 0.007842 | 0.008717 | 0.006418 | 0.008531 | 0.007432 |
| 11 August, 2017 | Foliage | 0.009274 | 0.008293 | 0.006316 | 0.011252 | 0.008097 |
| 25 August, 2017 | Foliage | 0.0082 | 0.008567 | 0.005991 | 0.008935 | 0.007321 |
| 8 September, 2017 | Foliage | 0.008008 | 0.007517 | 0.006073 | 0.009836 | 0.010445 |
| 22 September, 2017 | Foliage | 0.006842 | 0.006523 | 0.005484 | 0.008287 | 0.006503 |
| 6 October, 2017 | Foliage | 0.008566 | 0.007659 | 0.006422 | 0.009541 | 0.007612 |
| 3 November, 2017 | Foliage | 0.007438 | 0.006765 | 0.006127 | 0.009257 | 0.007271 |
| 17 November, 2017 | Foliage | 0.00925 | 0.007244 | 0.006076 | 0.00781 | 0.008178 |

**Table S5** Stomatal density of foliage data (number per cm^2^) in this study. ND = not determined.

| **Date** | **Type** | **Tree 1** | **Tree 2** | **Tree 3** | **Tree 4** | **Tree 5** |
| --- | --- | --- | --- | --- | --- | --- |
| 21 April, 2017 | Foliage | ND | ND | ND | ND | ND |
| 5 May, 2017 | Foliage | 55,659 | 57,013 | 48,815 | 60,172 | 57,013 |
| 19 May, 2017 | Foliage | 47,762 | 53,177 | 46,633 | 52,425 | 62,955 |
| 2 June, 2017 | Foliage | 52,277 | 48,454 | 41,223 | 54,771 | 67,902 |
| 16 June, 2017 | Foliage | 51,197 | 55,685 | 46,958 | 58,344 | 61,918 |
| 30 June, 2017 | Foliage | 57,098 | 47,124 | 50,615 | 67,154 | 71,393 |
| 14 July, 2017 | Foliage | 57,430 | 54,688 | 51,862 | 66,489 | 74,468 |
| 28 July, 2017 | Foliage | 57,763 | 54,688 | 45,130 | 59,924 | 66,905 |
| 11 August, 2017 | Foliage | 62,500 | 54,854 | 49,867 | 72,806 | 81,366 |
| 25 August, 2017 | Foliage | 60,422 | 53,939 | 52,111 | 56,932 | 72,390 |
| 8 September, 2017 | Foliage | 58,511 | 54,521 | 45,213 | 58,594 | 73,803 |
| 22 September, 2017 | Foliage | 60,339 | 49,867 | 47,789 | 61,420 | 71,061 |
| 6 October, 2017 | Foliage | 63,996 | 51,114 | 54,688 | 59,757 | 69,315 |
| 3 November, 2017 | Foliage | 54,189 | 54,854 | 47,872 | 67,985 | 70,645 |
| 17 November, 2017 | Foliage | 60,173 | 47,872 | 44,548 | 49,036 | 78,125 |

**Table S6** Total chlorophyll content of foliage (mg/g) in this study. ND = not determined.

| **Date** | **Type** | **Tree 1** | **Tree 2** | **Tree 3** | **Tree 4** | **Tree 5** |
| --- | --- | --- | --- | --- | --- | --- |
| 21 April, 2017 | Foliage | 0.961256 | 0.961256 | 0.787464 | 0.616495 | 0.691775 |
| 5 May, 2017 | Foliage | 1.457553 | 2.256827 | 2.343309 | 0.897177 | 0.877465 |
| 19 May, 2017 | Foliage | 1.682396 | 2.523567 | 2.102085 | 1.035390 | 1.498260 |
| 2 June, 2017 | Foliage | 1.997135 | 2.468951 | 3.253709 | 1.986754 | 2.779871 |
| 16 June, 2017 | Foliage | 2.769973 | 3.878431 | 4.237541 | 1.926254 | 3.160595 |
| 30 June, 2017 | Foliage | 2.709349 | 3.913021 | 4.025772 | 1.927592 | 3.072823 |
| 14 July, 2017 | Foliage | 2.277975 | 3.409191 | 3.833920 | 1.903710 | 2.848181 |
| 28 July, 2017 | Foliage | 2.200797 | 2.938170 | 4.325517 | 1.785965 | 3.218763 |
| 11 August, 2017 | Foliage | 2.393051 | 3.232026 | 3.961287 | 2.156760 | 2.724135 |
| 25 August, 2017 | Foliage | 2.450436 | 4.215970 | 4.692483 | 2.196292 | 2.349362 |
| 8 September, 2017 | Foliage | 2.244885 | 3.706012 | 4.520447 | 1.640695 | 2.500070 |
| 22 September, 2017 | Foliage | 2.288796 | 4.113555 | 4.134045 | 2.122550 | 2.542018 |
| 6 October, 2017 | Foliage | 1.924503 | 3.680643 | 3.674797 | 1.727037 | 2.347278 |
| 3 November, 2017 | Foliage | 1.225278 | 2.710124 | 2.540235 | 1.202815 | 1.315973 |
| 17 November, 2017 | Foliage | 0.080749 | 1.475312 | 0.535474 | 0.176889 | 0.254464 |

**Table S7** Total mercury (total Hg) content (in ng/g) in understory foliage and fresh litter of individual tree over the growing season.

| **Date** | **Type** | **Tree 1** | **Tree 2** | **Tree 3** | **Tree 4** | **Tree 5** |
| --- | --- | --- | --- | --- | --- | --- |
| 21 April, 2017 | Foliage | 6.2 | 7.1 | 8.3 | 3.9 | 7.6 |
| 5 May, 2017 | Foliage | 7.2 | 6.3 | 8.9 | 4.3 | 9.7 |
| 19 May, 2017 | Foliage | 11.1 | 9.1 | 9.8 | 6.1 | 12.7 |
| 2 June, 2017 | Foliage | 21.9 | 11.9 | 14.8 | 9.8 | 15.8 |
| 16 June, 2017 | Foliage | 19.4 | 10.9 | 18.0 | 12.1 | 20.6 |
| 30 June, 2017 | Foliage | 18.2 | 15.6 | 19.9 | 13.3 | 23.3 |
| 14 July, 2017 | Foliage | 25.0 | 17.5 | 20.4 | 16.0 | 14.3 |
| 28 July, 2017 | Foliage | 20.0 | 11.8 | 16.8 | 10.9 | 19.8 |
| 11 August, 2017 | Foliage | 17.7 | 18.2 | 27.4 | 13.9 | 25.1 |
| 25 August, 2017 | Foliage | 27.4 | 26.2 | 29.7 | 20.3 | 47.5 |
| 8 September, 2017 | Foliage | 28.2 | 23.5 | 32.2 | 18.5 | 27.6 |
| 22 September, 2017 | Foliage | 30.0 | 17.3 | 30.8 | 15.3 | 47.4 |
| 6 October, 2017 | Foliage | 24.4 | 25.7 | 28.0 | 21.8 | 35.3 |
| 3 November, 2017 | Foliage | 38.9 | 34.2 | 40.8 | 30.4 | 48.1 |
| 17 November, 2017 | Foliage | 40.6 | 35.1 | 42.8 | 33.4 | 38.9 |
| 28 November, 2017 | Fresh litter | 50.1 | 32.3 | 58.0 | 35.6 | 58.3 |

**Table S8** Methylmercury (MeHg) content (in pg/g) in understory foliage and fresh litter of individual tree over the growing season.

| **Date** | **Type** | **Tree 1** | **Tree 2** | **Tree 3** | **Tree 4** | **Tree 5** |
| --- | --- | --- | --- | --- | --- | --- |
| 21 April, 2017 | Foliage | 22.1 | 69.9 | 27.6 | 17.9 | 72.7 |
| 5 May, 2017 | Foliage | 13.8 | 30.8 | 36.0 | 12.8 | 59.1 |
| 19 May, 2017 | Foliage | 21.4 | 27.3 | 16.8 | 8.3 | 66.7 |
| 2 June, 2017 | Foliage | 21.7 | 13.9 | 14.8 | 11.6 | 66.0 |
| 16 June, 2017 | Foliage | 25.1 | 16.5 | 21.1 | 9.0 | 41.8 |
| 30 June, 2017 | Foliage | 23.4 | 15.2 | 28.6 | 14.1 | 29.8 |
| 14 July, 2017 | Foliage | 29.0 | 15.3 | 30.4 | 15.4 | 21.7 |
| 28 July, 2017 | Foliage | 17.8 | 24.7 | 35.1 | 25.3 | 13.5 |
| 11 August, 2017 | Foliage | 26.6 | 13.1 | 42.5 | 9.3 | 16.6 |
| 25 August, 2017 | Foliage | 25.1 | 16.9 | 28.4 | 16.9 | 16.2 |
| 8 September, 2017 | Foliage | 31.3 | 7.1 | 30.4 | 20.1 | 31.8 |
| 22 September, 2017 | Foliage | 34.8 | 14.2 | 26.3 | 8.2 | 42.8 |
| 6 October, 2017 | Foliage | 36.6 | 36.0 | 38.9 | 18.5 | 43.5 |
| 3 November, 2017 | Foliage | 26.1 | 29.1 | 47.3 | 27.9 | 32.8 |
| 17 November, 2017 | Foliage | 40.9 | 35.2 | 47.7 | 50.2 | 32.7 |
| 28 November, 2017 | Fresh litter | 34.2 | 29.2 | 44.2 | 22.2 | 40.2 |

**Table S9** Percentage of total mercury as methylmercury (%MeHg) in understory foliage and fresh litter of individual tree over the growing season.

| **Date** | **Type** | **Tree 1** | **Tree 2** | **Tree 3** | **Tree 4** | **Tree 5** |
| --- | --- | --- | --- | --- | --- | --- |
| 21 April, 2017 | Foliage | 0.36 | 0.98 | 0.33 | 0.46 | 0.96 |
| 5 May, 2017 | Foliage | 0.19 | 0.49 | 0.40 | 0.30 | 0.61 |
| 19 May, 2017 | Foliage | 0.19 | 0.30 | 0.17 | 0.14 | 0.53 |
| 2 June, 2017 | Foliage | 0.10 | 0.12 | 0.10 | 0.12 | 0.42 |
| 16 June, 2017 | Foliage | 0.13 | 0.15 | 0.12 | 0.07 | 0.20 |
| 30 June, 2017 | Foliage | 0.13 | 0.10 | 0.14 | 0.11 | 0.13 |
| 14 July, 2017 | Foliage | 0.12 | 0.09 | 0.15 | 0.10 | 0.15 |
| 28 July, 2017 | Foliage | 0.09 | 0.21 | 0.21 | 0.23 | 0.07 |
| 11 August, 2017 | Foliage | 0.15 | 0.07 | 0.16 | 0.07 | 0.07 |
| 25 August, 2017 | Foliage | 0.09 | 0.06 | 0.10 | 0.08 | 0.03 |
| 8 September, 2017 | Foliage | 0.11 | 0.03 | 0.09 | 0.11 | 0.12 |
| 22 September, 2017 | Foliage | 0.12 | 0.08 | 0.09 | 0.05 | 0.09 |
| 6 October, 2017 | Foliage | 0.15 | 0.14 | 0.14 | 0.08 | 0.12 |
| 3 November, 2017 | Foliage | 0.07 | 0.09 | 0.12 | 0.09 | 0.07 |
| 17 November, 2017 | Foliage | 0.10 | 0.10 | 0.11 | 0.15 | 0.08 |
| 28 November, 2017 | Fresh litter | 0.07 | 0.09 | 0.08 | 0.06 | 0.07 |

**Table S10** Statistical analysis of the linear regression relationship between total-Hg content in understory foliage and different factors, including average temperature (Temp.) two weeks prior to the sampling date, average evapotranspiration (ET) two weeks prior to the sampling date, total amount of precipitation two weeks prior to the sampling date, average solar radiation (Solar) two weeks prior to the sampling date, foliar total chlorophyll (Total chl.), and foliar stomatal density. NS= not significant (*p*>0.05).

|  | Temp. | ET | Precip. | Solar | Total chl. | Stomatal density |
| --- | --- | --- | --- | --- | --- | --- |
| Tree 1 | NS | -0.0044 | NS | -0.006 | NS | NS |
| Tree 2 | NS | -0.0015 | NS | -0.0036 | NS | NS |
| Tree 3 | NS | -0.0017 | NS | -0.0035 | NS | NS |
| Tree 4 | NS | -0.0015 | NS | -0.0031 | NS | NS |
| Tree 5 | NS | -0.0039 | NS | -0.0095 | NS | +0.034 |

**Table S11** Statistical analysis of the linear regression relationship between MeHg content in understory foliage and different factors, including average temperature (Temp.) two weeks prior to the sampling date, average evapotranspiration (ET) two weeks prior to the sampling date, total amount of precipitation two weeks prior to the sampling date, average solar radiation (Solar) two weeks prior to the sampling date, foliar total chlorophyll (Total chl.), and foliar stomatal density. NS= not significant (*p*>0.05).

|  | Temp. | ET | Precip. | Solar | Total chl. | Stomatal density |
| --- | --- | --- | --- | --- | --- | --- |
| Tree 1 | NS | -0.020 | NS | NS | NS | +0.043 |
| Tree 2 | NS | NS | NS | NS | -0.001 | NS |
| Tree 3 | NS | -0.020 | NS | -0.022 | NS | NS |
| Tree 4 | +0.010 | -0.003 | NS | -0.001 | -0.015 | NS |
| Tree 5 | NS | NS | NS | NS | NS | +0.014 |
